# A Prospective Study Assessing the Post-Prostatectomy Detection Rate of a Presumed Local Failure at mpMR with Either ^64^CuCl_2_ or ^64^CuPSMA PET/CT

**DOI:** 10.3390/cancers13215564

**Published:** 2021-11-06

**Authors:** Adriana Faiella, Rosa Sciuto, Diana Giannarelli, Marta Bottero, Alessia Farneti, Luca Bertini, Sandra Rea, Valeria Landoni, Patrizia Vici, Maria Consiglia Ferriero, Giuseppe Sanguineti

**Affiliations:** 1 Department of Radiation Oncology, IRCCS Regina Elena National Cancer Institute, 00144 Rome, Italy; adriana.faiella@ifo.gov.it (A.F.); marta.bottero@ifo.gov.it (M.B.); alessia.farneti@ifo.gov.it (A.F.); 2 Department of Nuclear Medicine, IRCCS Regina Elena National Cancer Institute, 00144 Rome, Italy; rosa.sciuto@ifo.gov.it (R.S.); sandra.rea@ifo.gov.it (S.R.); 3 Department of Biostatistics, IRCCS Regina Elena National Cancer Institute, 00144 Rome, Italy; diana.giannarelli@ifo.gov.it; 4 Department of Radiology, IRCCS Regina Elena National Cancer Institute, 00144 Rome, Italy; luca.bertini@ifo.gov.it; 5 Department of Physics, IRCCS Regina Elena National Cancer Institute, 00144 Rome, Italy; valeria.landoni@ifo.gov.it; 6 Department of Phase IV Clinical Experimentation, IRCCS Regina Elena National Cancer Institute, 00144 Rome, Italy; patrizia.vici@ifo.gov.it; 7 Department of Urology, IRCCS Regina Elena National Cancer Institute, 00144 Rome, Italy; mariaconsiglia.ferriero@ifo.gov.it

**Keywords:** prostate cancer, salvage radiotherapy, PET/CT, multiparametric MR

## Abstract

**Simple Summary:**

The role of PET/CT with two novel tracers was investigated in prostate cancer patients with both a biochemical failure after surgery and a presumed local failure at multiparametric MR. Overall, both PET tracers detected only about 50% of local failures. Therefore, multiparametric MR remains the exam of choice to investigate the prostatic fossa in patients who fail surgery.

**Abstract:**

Background: We aimed assess the detection rate (DR) of positron emission tomography/computed tomography with two novel tracers in patients referred for salvage radiotherapy (sRT) with a presumed local recurrence at multiparametric magnetic resonance (mpMR) after radical prostatectomy (RP). Methods: The present prospective study was conducted at a single institution between August 2017 and June 2020. Eligibility criteria were undetectable PSA after RP; subsequent biochemical recurrence (two consecutive PSA rises to 0.2 ng/mL or greater); a presumed local failure at mpMR; no distant metastases at ^18^F-fluorocholine PET/CT (CH/PET); no previous history of androgen deprivation therapy. Patients were offered both ^64^CuCl_2_ PET/CT (CU/PET) and ^64^Cu-PSMA PET/CT (PSMA/PET) before sRT. After image co-registration, PET findings were compared to mpMR ones in terms of DR and independent predictors of DR investigated at logistic regression. Results: A total of 62 patients with 72 nodules at mpMR were accrued. Compared to mpMR (DR = 100%, 95%CI: 94.9–100%), DRs were 47.2% (95%CI: 36.1–58.6%) and 54.4% (95%CI: 42.7–65.7%) for CU/PET and PSMA/PET, respectively (*p* < 0.001 for both). Both experimental PET/CT performed particularly poorly at PSA levels consistent with early sRT. Conclusions: The two novel radiotracers are inferior to mpMR in restaging the prostatic fossa for sRT planning purposes, particularly in the context of early salvage radiotherapy.

## 1. Introduction

Despite radical prostatectomy (RP), up to 50% of the patients with high-risk prostate cancer (PCa) will experience biochemical failure [1]. Salvage radiotherapy (sRT) can be the only curative option, especially when the disease is confined to the pelvis [2]. The purpose of re-staging in the setting of biochemical failure after RP is twofold: to rule out systemic disease and to try to localise areas of macroscopic disease for which a higher dose of radiation is desirable [3]. Currently, multiparametric MR (mpMR) is most frequently used to assess the primary tumor site, while whole-body PET/CT of both N and M sites [3]. However, ideally, re-staging should be performed with a single imaging procedure. The “MRI-only” approach combining mpMR to assess the prostatic bed and whole-body MR for the rest of the body has been shown to be feasible [4], although it is hampered by the complexity of whole-body MR [3]. Alternatively, the “PET/CT-only” approach has been limited by the poorer sensitivity to detect local disease of choline PET/CT over mpMR [5,6]. Novel radiotracers may provide improved sensitivity for the detection of local disease due to increased avidity of uptake and a more favorable lesion-to-background ratio [3], but no data are currently available against mpMR in the context of sRT. Therefore, the aim of the present study was to prospectively assess the (local) performance of PET/CT with two novel radiotracers compared to the gold standard (mpMR) in the setting of biochemical failure after prostatectomy.

## 2. Materials and Methods

### 2.1. Patients

The present IRB approved prospective study (RS 946/17) was conducted at a single Institution between January 2017 and June 2020 (Clinicaltrials.gov NCT04703543). All patients provided specific written informed consent. Patients had to preliminarily satisfy all the following eligibility criteria: history of localised prostate cancer treated with RP; undetectable (<0.1 ng/mL) PSA after RP; subsequent biochemical recurrence (2 consecutive PSA rises to 0.2 ng/mL or higher) [7]; a presumed local failure at mpMR (as defined below); no distant metastases at restaging choline-PET/CT (CH/PET); no previous history of androgen deprivation therapy and/or radiotherapy. As part of the research protocol, accrued patients were offered both ^64^CuCl_2_-PET/CT (CU/PET) and ^64^Cu-PSMA-PET/CT (PSMA/PET) before being considered for sRT. The structure of ^64^Cu-PSMA-617 is reported in the Supplementary Material (Figure S1).

### 2.2. Scans

All scans were obtained at our Institution. Patients were scanned and treated with a half empty/half full bladder and an empty rectum. Multiparametric MR was performed on a 3T system (Discovery MR750; General Electric) equipped with surface phased array coil. MpMR consisted of a DCE (dynamic contrast-enhanced) sequence involving a 3D LAVA (liver acquisition with volume acceleration) gradient echo sequence, with repetition time/echo time 5.4–5.9/1.6–1.7 ms, flip angle 15°, acquisition matrix 320 × 192, field of view 34 cm, and slice thickness 3 mm. Dynamic volumes were acquired with a temporal resolution of 10–16 s depending on the length of the scan, which was correlated to the patient anatomy, for a total of 20 phases/acquisition (scan time ≈5 min). At the third dynamic volume, 0.1 mmol/kg body weight of gadobutrol (Gadovist; Bayer) was administered intravenously at a rate of 3 mL/s. T2w axial, coronal, and sagittal plane sequences were also performed to obtain precise anatomical site of the DCE findings. DWI sequence was also included with b-values of 0, 500, 1000, and 2000 s/mm^2^.

All PET/CT scans were performed on a Biograph 16 tomograph (Siemens Medical System, Erlangen, Germany). An average dose of 5 MBq/kg of ^18^F-fluorocholine, ^64^Cu-Cl_2_, or ^64^Cu-PSMA was administered intravenously 60 ± 10 min before image acquisition, respectively, in CH/PET, CU/PET, and PSMA/PET. A non-contrast-enhanced whole-body CT scan was acquired for anatomic localisation and attenuation correction (120–140 Kv, 4 mm slice thickness). Whole-body PET was acquired in 3D mode immediately after the CT scan, 3 or 4 min for each bed position for CH/PET and CU/PET-PSMA/PET, respectively. A late acquisition of the pelvis was also performed 2 h after radiotracer injection and 1 h after i.v. injection of 20 mg furosemide for CH/PET and PSMA/PET. PET images were reconstructed by the TrueX algorithm (Siemens Medical System, Erlangen, Germany) with point spread function modelling, using 3 iterations and 21 subsets. After reconstruction, the images were filtered by a Gaussian filter with a full width at half maximum of 4 mm. PET/CT images were reviewed and analysed using the Syngo.via software (Siemens Medical System, Erlangen, Germany).

All imaging studies (mpMR and all nuclear medicine studies) had to be completed within 30 days.

### 2.3. Image Reading and Evaluation

All mpMR scans were interpreted by a single observer (L.B.) as per clinical practice. A presumed local failure at mpMR was considered as an early/fast enhancing discrete lesion on DCE-MRI, possibly accompanied by a hyperintense soft tissue on T2w [8]. The detection of a hyperintense focus on DWI in the site of a positive DCE sequence empowered the diagnosis. Abnormalities seen exclusively on T2w images were disregarded. No pathologic confirmation was obtained. The same observer contoured each presumed lesions in the prostatic fossa (and seminal vesicles bed) using a manual delineation tool of MIM software (version 6.9.4, MIM Software Inc., Beachwood, OH, USA). The location of each lesion was classified as per Connolly et al. in perianastomotic (posterior, lateral, anterior), bladder neck, and retrovesical [9]. The maximum axial diameter (mm) of the nodule along with its volume was extracted for each visible lesion. CH/PET studies were evaluated by one nuclear medicine physician during routine clinical practice as previously reported [10]. CU/PET and PSMA/PET were jointly reviewed by two experienced nuclear medicine physicians (R.S. and S.R.) trained in copper PET reading. The two observers were aware of the ‘positivity’ of the mpMR scan but not of the number and the location of the presumed local failure(s). Depending on the detection of 1 (or more) area(s) of focal non-physiological uptake in the prostatic fossa, the PET/CT study was preliminarily defined as positive or negative. Moreover, each presumed positive lesion at CH/PET, CU/PET, or PSMA/PET was jointly contoured in MIM software using as threshold the 50% of the maximum SUV value [11,12].

### 2.4. Co-Registration and Transfer of Image Findings to the Planning CT

Once the lesion(s) had been contoured on mpMR, T2w images were co-registered to the planning CT (plCT) by a medical physicist under the supervision of the treating radiation oncologist using MIM software tools (Figure S2). Indeed, the process allowed for the identification of a higher dose volume on plCT that was exploited for radiotherapy planning purposes and clinical treatment. A rigid co-registration procedure focusing on the volume harbouring the nodule was preliminary run using box-based alignment tools. Particular attention was placed on the relationships between the nodule and the immediate surrounding structures (pelvic bone, the rectum/bladder walls, and other reference points such as calcifications; Figure S2). In case of significant deformation (i.e., bladder filling), the fusion could be locally deformed using the ‘Reg refine’ tool from MIM. Once the rigid registration was optimised, the contour of each identified lesion was transferred from the mpMR to the plCT. A similar approach (medical physicist/radiation oncologist, rigid and eventually locally deformed) was used for co-registering selected PET/CT to the corresponding plCT. The process involved only the scans with the lesions previously contoured by nuclear medicine physicians. The rigid registration was manually adjusted as described above, between the plCT and the CT of each available PET/CT. For a same-modality co-registration (CT-to-CT) and for such small volume ROIs, anatomic landmarks (i.e., posterior bladder wall, anterior rectal wall) as well as other reference points (i.e., surgical clips, calcifications) close to the lesion were exploited to refine the co-registration process. Again, the Reg Refine tool of MIM was used in selected cases to further improve the co-registration around the ROI. Once the co-registration between plCT and CT/PET had been optimised, available contours were transferred to the plCT as well.

### 2.5. Statistical Analysis

**Figure 1:**
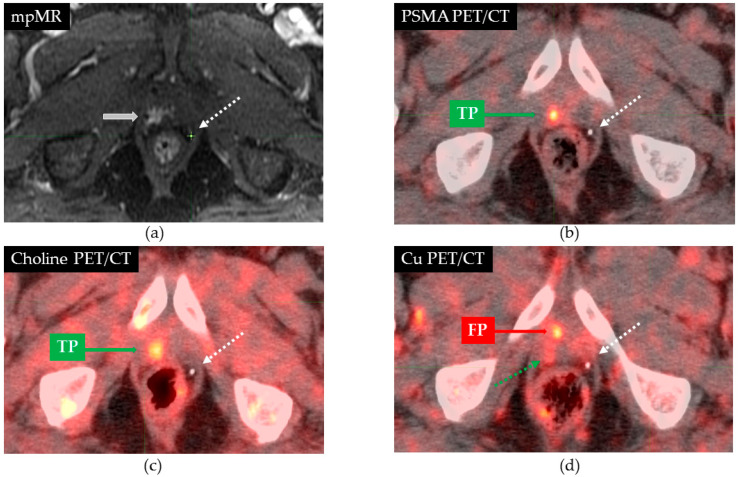
Axial slices showing the position of the nodule on the various imaging modalities: mpMR (**a**), PSMA/PET (**b**), CH/PET (**c**), and CU/PET (**d**). On multiparametric MR, the nodule is indicated by the thick white arrow. A focal lesion is shown on all PET/CT scans, while the dashed white arrow indicates the position of a calcification. The distances between the centroid on mpMR and PSMA PET/CT, Cu PET/CT, and choline PET/CT were 0.43, 1.21, and 0.47 cm, respectively. Therefore, lesions seen on both PSMA and choline PET/CT were considered true positives (TP), while the one on CU PET/CT was a false positive. Interestingly, on Cu PET/CT, a blurred mild uptake was detected in the area that would correspond to the TP lesion on mpMR (green dashed line).

Once the plCT harboured all the available contours (those form the mpMR as well as those detected at any PET/CT), the position (*x, y, z*) of the centroid of each ROI was extracted, and the distance between each PET/CT ROI and the plCT ROI was calculated. We arbitrarily defined PET/CT lesions whose centroid was within 1 cm from the corresponding one at mpMR as true positives (TP). Otherwise, contoured ROIs were defined as false positives (FP). The co-registration process for FP lesions whose centroid was within 2 cm from the one at mpMR was retrospectively reviewed and further optimised as best as possible. The ‘best’ or ‘minimum’ centroid distance is reported. An example of TP and FP lesions is provided in Figure 1. In the case of multiple lesions in the same patient, each nodule was evaluated and treated separately. Therefore, the co-registration process was optimised for each nodule. Among FP, we further distinguished those lesions who did not have a counterpart at mpMR, or, in other words, were outnumbered (ON) at PET/CT.

The detection rate (DR) corresponds to the proportion of lesions that are detected with a given imaging modality. Univariable binary logistic regression analyses on DR for each of the PET tracers were performed considering Gleason Grade Grouping at RP (1–2 vs. 3 vs. 4–5), PSA value at failure (continuum), PSA doubling time at failure (continuum), the nodule volume at mpMR (continuum) and the location of the failure (anastomotic vs. bladder neck vs. retrovesical). Regarding the PSA level at failure for patients with multiple lesions, the PSA value assigned to each nodule was obtained after rescaling the total PSA value by the proportional volume of the contributing nodules (individual weighted PSA-iwPSA).

Covariates with *p*-value ≤ 0.2 at univariable analysis were entered into a multivariable model. Upper and lower 95% confidence interval limits for the logistic function were calculated on the basis of the standard error of the linear logistic predictor. DRs were compared with the McNemar test for correlated proportions. Uncorrelated proportions were compared with the chi-squared test. Individual matched pairs were compared with the Wilcoxon signed rank test. Confidence intervals (CI) for proportions were computed with the Wilson score method without continuity correction. As per current clinical guidelines, no pathological confirmation of presumed local failures at mpMR was obtained. Since mpMR is the current gold standard to diagnose (and contour) local failures [3], the present study was intended to test how PET/CT would perform with respect to mpMR. Assuming a DR of PET/CT up to 80%, the analysis of 60 patients/60 nodules would have allowed the estimation of DR with a 95%CI half-width of 10%. All tests were performed two-sided, and statistical significance was claimed for *p*-values <0.05. All statistical tests were performed using GraphPad (version 8.0.1, GraphPad Software Inc., San Diego, CA, USA) and SPSS (version 25, IBM, Armonk, NY, USA).

## 3. Results

During the study period, 277 patients were referred for consideration of sRT following biochemical failure after RP. Of them, 215 (77.6%) were excluded, as detailed in the CONSORT diagram (Figure S3). One patient was re-staged with whole-body MR instead of choline PET/CT, but he was deemed to be eligible for the protocol. Four patients underwent only CH/PET and CU/PET, but refused PSMA/PET. They were kept in the analysis. Therefore, the number of available imaging studies were 62, 62, 58, and 61 for mpMR, CU/PET, PSMA/PET, and CH/PET, respectively. Selected characteristics are reported in Table 1.

Consistently with the inclusion criterion, mpMR described a presumed local failure in all 62 patients (DR = 100%, 95%CI: 94.9–100%). Nine patients were found to have multiple local failures (two nodules, 8 pts; three nodules, 1 pt) for a final number of 72 lesions. The location of each lesion within the prostatic fossa is reported in Table 2.

### 3.1. PET/CT Findings

The number of all discrete lesions detected within the prostatic fossa at PET/CT were 26, 50, and 43 for choline, Cu, and PSMA, respectively (Table 3).

**Figure 2:**
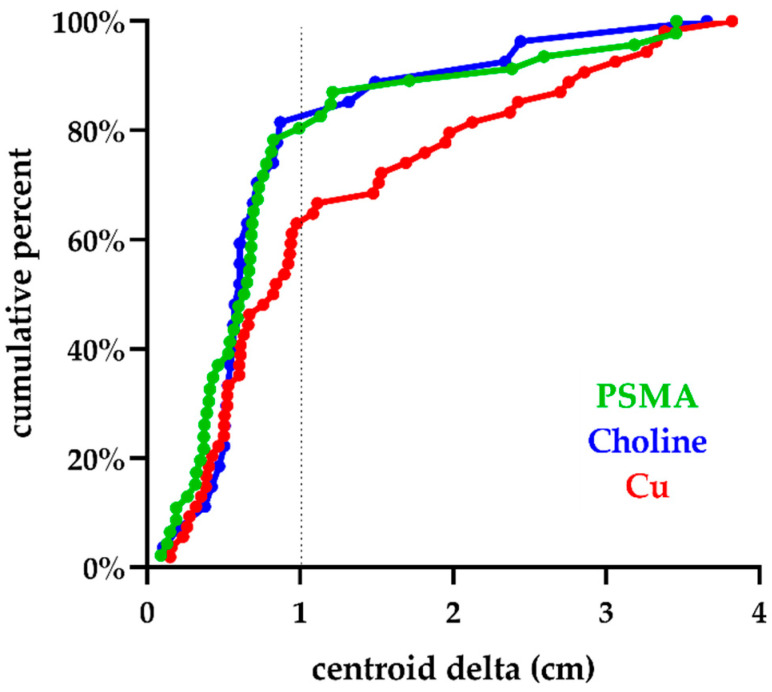
Cumulative frequency of minimum centroid distance for each tracer.

At both CU/PET and PSMA/PET, one patient was found to have an additional nodal failure within the pelvis that had not been seen at CH/PET, while no distant metastases were discovered by any PET/CT tracer. Out of all detected lesions, the numbers of TP lesions were 22 (84.6%), 34 (68.0%), and 37 (86.0%) for CH/PET, CU/PET, and PSMA/PET, respectively (Table 3). We observed a significantly higher rate of FP for CU/PET (16/50, 32.0%) over PSMA/PET (6/43, 14.0%, *p* = 0.041). Among FP, ON lesions were one (25.0%), four (25.0%), and three (50.0%) for CH/PET, CU/PET, and PSMA/PET, respectively. Overall, the mean (SD) minimum distances between the centroid of mpMR and PET/CT lesions were 0.87 (0.78), 1.21 (1.00), and 0.86 (0.84) cm for CH/PET, CU/PET, and PSMA/PET, respectively. The cumulative frequency of minimum centroid distance for each tracer is shown in Figure 2.

**Figure 3:**
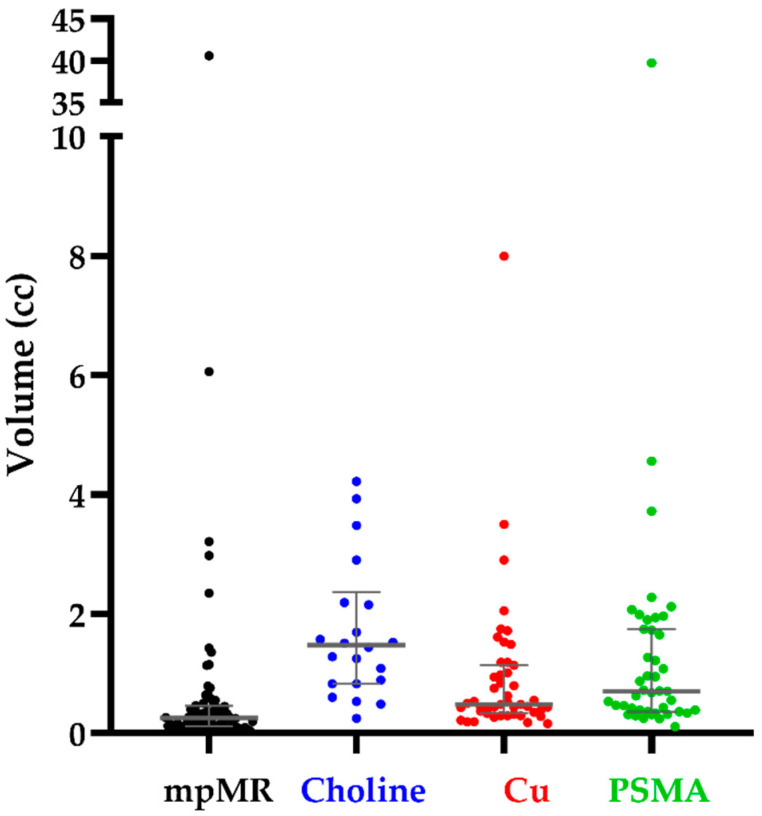
Individual volumetric data TP lesions at mpMR and PET/CT. Grey lines indicate the median values along with the interquartile range.

Individual volumetric data are illustrated in Figure 3. Mean (SD) volumes were 1.06 (4.81) cc, 1.03 (1.41) cc, 2.08 (6.44) cc, and 2.44 (3.88) at mpMR, CU/PET, PSMA/PET, and CH/PET, respectively (mpMR vs. PSMA/PET, 37 pairs, *p* = 0.011; mpMR vs. CU/PET, 34 pairs, *p* = 0.462; mpMR vs. CH/PET, 22 pairs, *p* = 0.372).

### 3.2. Detection Rates at PET/CT

**Figure 4:**
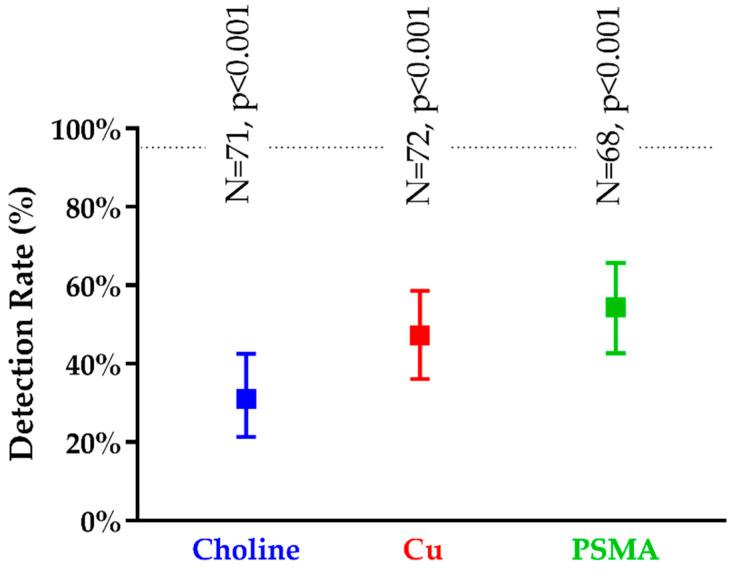
Detection rates (95%CI) of true positive lesions for PET/CTs. The number indicates the detectable lesions for each imaging modality. The *p*-value refers to the paired comparison of a given PET/CT with mpMR. The horizontal dashed black line corresponds to the lower 95%CI of DR for mpMR.

As illustrated in Figure 4, the DRs of TP lesions were 31.0% (95%CI: 21.4–42.5%), 47.2% (95%CI: 36.1–58.6%), and 54.4% (95%CI: 42.7–65.7%) for CH/PET, CU/PET, and PSMA/PET, respectively. All PET/CT performed significantly worse than mpMR (*p* < 0.001 for all). Both CU/PET and PSMA/PET showed a higher rate of local disease detection than CH/PET (*p* < 0.001 for both). Conversely, CU/PET and PSMA/PET did not perform differently from each other (*p* = 0.424).

### 3.3. Subgroup Analysis

Results of univariable analyses are shown in Table S1. Median iwPSA was 0.40 ng/mL (IQR: 0.205–0.600 ng/mL). Both the volume of the lesion at mpMR and the iwPSA were consistently correlated to DRs for all tracers at UV analysis. The two covariates were poorly correlated with each other (Rho = 0.44, *p* < 0.001). Other covariates had a variable impact on each tracer, as shown in Table S1. For MV analysis, lesions with GLS 4 + 3 and higher were pooled, as well as lesions above the anastomosis.

The volume of the nodule at mpMR was the only independent predictor of TP for choline (for every 0.1 cc, OR = 1.974, 95%CI: 1.324–2.943, *p* = 0.001). Regarding CU/PET, both iwPSA and PSADT were independently correlated to DR (OR = 5.710, 95%CI: 1.389–23.480, *p* = 0.016, and OR = 0.954, 95%CI: 0.910–1.000, *p* = 0.048 for iwPSA and PSADT, respectively). When iwPSA was replaced by PSA, the odds ratio for PSA decreased (OR = 3.763, 95%CI 1.085–13.053, *p* = 0.037) and PSADT became marginally significant (*p* = 0.058).

**Figure 5:**
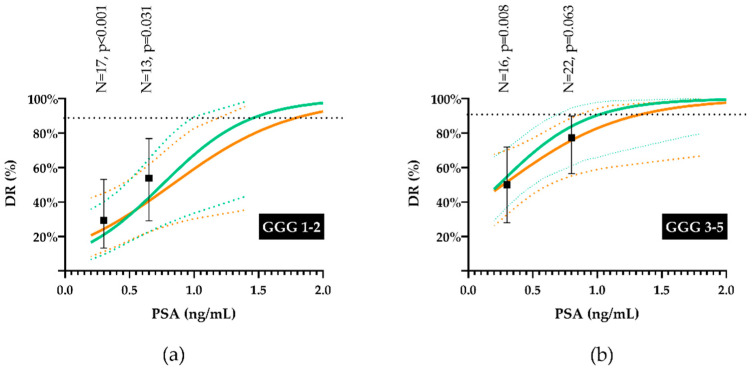
Estimated DR according to the logistic function for PSMA PET/CT by Gleason Grade Group (GGG): GGG 1-2 (**a**) and GGG 3-5 (**b**). The green continuous and the orange continuous lines correspond to individual weighted PSA and total serum PSA, respectively. Dashed lines identify the 95% confidence intervals of the logistic function. The horizontal dashed black line is the lower 95%CI of DR at mpMR. The black dots (and 95%CI bars) correspond to the observed DR after stratification of nodules by median iwPSA.

The iwPSA value at failure (OR = 18.687, 95%CI: 2.149–162.5, *p* = 0.008) and GGG (GGG3-5 vs. GGG1-2, OR = 4.532, 95%CI: 1.456–14.11, *p* = 0.009) were independent predictors of DR for PSMA/PET. As shown in Figure 5, the performance of PSMA/PET was poor at PSA levels below the observed median iwPSA values (<0.4 ng/mL), particularly for GGG1-2 lesions. The performance of PSMA PET/CT was significantly inferior to the one of mpMR up to iwPSA values of ≈1.0 ng/mL and ≈0.6 ng/mL for GGG1-2 and GGG3-5 lesions, respectively. The analysis was repeated replacing iwPSA with the total serum PSA at failure. Both PSA and GGG were confirmed to be independent predictors of DR at MV analysis, although the OR for PSA halved (OR = 8.583, 95%CI: 1.295–56.901, *p* = 0.026 and OR = 3.317, 95%CI: 1.111–9.904, *p* = 0.032 for PSA and GGG, respectively). Moreover, as illustrated in Figure 5, the use of total serum PSA (orange line) in place of iwPSA (green line) shifted the curve to the right at high PSA levels, increasing the PSA threshold at which PSMA PET/CT and mpMR do not perform differently (from ≈1.0 ng/mL to ≈1.2 ng/mL and from ≈0.6 ng/mL to ≈0.8 ng/mL for GGG1-2 and GGG3-5 lesions, respectively).

## 4. Discussion

In the setting of biochemical failure after radical prostatectomy without extrapelvic disease at choline PET/CT and with a presumed local failure at mpMR, the present study shows that both PSMA/PET and CU/PET failed to localise a significant number of local lesions compared to mpMR. Importantly, the performance of PET/CT was particularly poor in the subgroup of patients who undergo early salvage radiotherapy. Since patients with a biochemical failure after RP undergo re-staging to rule out systemic disease before sRT [13], it would be desirable to have a diagnostic test that also addresses the presence of local disease. The identification along with the precise localisation of the disease within the prostatic fossa is supported by both the European Society Urologic Oncology and the American College of Radiology [14,15] since it may have prognostic value [16,17] and it may also optimise both sRT dose prescription and planning [18,19], although the role of a MR-directed boost to macroscopic disease remains undetermined.

Multiparametric MR is the most accurate imaging method to identify local recurrence after RP with sensitivity and specificity rates ranging from 84 to 100% and 89 to 97%, respectively [15]. Despite an estimated rate of false positives up to 10% [6,8], mpMR remains the gold standard for the diagnosis of local recurrence after RP, and biopsy is generally considered unnecessary before sRT. On the other hand, it is well documented that CH/PET has a poor local DR, especially when it is radiolabeled with ^18^F, whose urinary excretion interferes with prostatic bed imaging [3,6]. Moreover, the performance of CH/PET is strictly correlated to the volume of the disease (Table S1), and current guidelines recommend sRT at PSA values [20] well below the detectability of CH/PET.

Several novel PET probes have been assessed for their ability to detect PCa recurrence [21]. Of them, ^18^F fluciclovine and ^68^Ga PSMA have gained acceptance in the USA and in Europe, respectively, due to their higher sensitivity than F-choline, especially at lower PSA levels [22,23]. To date, the few available studies directly comparing mpMR and PSMA-ligand PET/CT in recurrent PCa provide inconsistent results, showing either similar [24] or super local DR for ^68^Ga PSMA PET/CT [25,26]. Therefore, in the present paper, we investigated whether novel copper PET/CT (^64^CuCl_2_ or ^64^Cu-PSMA) [27] can replace mpMR for local restaging after RP. Copper 64 (^64^Cu) can be used alone in the form of ^64^CuCl_2_ or labelled to PSMA (^64^Cu-PSMA). The former targets copper metabolism and replicates copper fluxes and bio-distribution. An imbalance in copper metabolism plays an important role in cancer development and growth, particularly in tumour angiogenesis and endothelial cell proliferation [28]. Moreover, preclinical studies have shown an increased ^64^CuCl_2_ uptake [29] as well an over-expression of human copper transporter 1 (hCTR1) [30] in prostate cancer cells. Therefore, ^64^Cu has been proposed as a PET radiotracer to target cancer-related copper metabolism alterations of human prostate cancer [31], although clinical data are scarce and inconclusive [32,33]. When copper is used to radiolabel PSMA, its biomolecular target becomes the prostate-specific membrane antigen, a membrane-bound glycoprotein significantly over-expressed in prostate cancer cells. To date, many PSMA ligands have been tested, including ^68^Ga-PSMA, ^64^Cu PSMA, ^18^F-DCFPyl PSMA, and ^18^F-PSMA. PSMA-617 presents one of the highest binding affinities to the PSMA receptor that have been published thus far [34,35]. A recent meta-analysis comparing different PSMA tracers in the restaging of biochemical failure of prostate cancer after definitive treatment concluded that no PSMA tracer can be currently considered superior to others [36]. Compared to ^68^Ga, ^64^Cu has the theoretical advantages of a longer half-life, allowing later acquisitions and a shorter positron range providing higher spatial resolution. Therefore, ^64^Cu PSMA should be considered at least equivalent to ^68^Ga-PSMA in the location of disease after post-prostatectomy biochemical failure. On average, the local DRs obtained with ^64^CuCl_2_ and ^64^CuPSMA PET/CT were very similar to each other and to those reported by others [30,32].

The present study, which prospectively compared mpMR and PET/CT with various tracers for local re-staging after RP within a relatively homogenous patient population with contemporary PSA values, shows that none of the PET/CT tracers was able to perform as mpMR in detecting and localising the site of the presumed local disease. Instead of using biopsy results or oncologic outcomes after sRT, we validated PET/CT findings against mpMR ones after anatomic co-registration of the presumed local lesions that are features relevant to sRT [18]. Since mpMR is routinely used to detect local failures for planning purposes at our institution and elsewhere [8,18], the present study answers the question as to whether PET/CT might replace mpMR in the detection and the delineation of local failures for sRT planning purposes. Of note, as illustrated in Figure 5, the performance of PSMA/PET approaches the one of mpMR only at PSA levels that are significantly higher than the ones recommended at sRT.

Three methodological aspects of the present study deserve further discussion. First, detection and contouring of PET/CT lesions involved two senior nuclear medicine physician who jointly reviewed the scans blindly to mpMR findings but the fact that the scan had to contain at least one local lesion. Therefore, we cannot exclude that this leaned towards the detection of a higher-than-expected number of local lesions at PET/CT, which would be a bias favouring rather than limiting the performance of PET/CT. For the same reason, the rate of FP at PET/CT should be interpreted with caution since it might have been overestimated. Regarding contouring lesions at PET/CT, several delineation solutions have been proposed, although the optimal methodology for contouring target lesions has not yet been definitely established [37,38]. A popular method is a semi-automatic segmentation of the target lesion based on a fixed threshold that is commonly 40–43% of the SUVmax for 18F-FDG [39] and 30–50% for PSMA [11,12,40], although no specific data are available in the setting of early local recurrence of prostate cancer. Interestingly, as shown in Figure 3, the average volumetric results between CU/PET and PSMA/PET were markedly different, suggesting that the same threshold may not be appropriate for all tracers. However, since the centroid of the lesion rather than its volume was crucial to differentiate between TP and FP lesions, we do not believe that the threshold used for contouring had a major impact on results. The second aspect involves the co-registration of diagnostic images to the plCT. For all co-registrations, we started from a rigid deformation approach with the option to further optimise it with the Reg Refine tool from MIM vista to account for local deformations [41]. Moreover, in order to distinguish between TP and FP lesions, we arbitrarily set the tolerance of the displacement of paired centroids at 1 cm to account for uncertainties. This would also be consistent with the 5 mm margin we add isotropically to the CTV to define the PTV, considering that two co-registration processes (from mpMR to plCT and from PET/CT to plCT) were performed. Finally, we revised co-registrations that had led to borderline results (displacements from 1 to 2 cm). However, even in this case, we do not believe that this aspect had a major impact on results, since, even considering all discrete lesions seen at PET/CT as positive ones (Table 3), DRs would have still been significantly inferior for any tracer to the ones achieved by mpMR (*p* < 0.001 for all tracers). Third, we investigated predictors of DR in order to try to identify a subgroup of patients for whom either PSMA or Cu PET/CT may be a valid alternative to mpMR, but this was the case only for PSA values outside current recommendations. We investigated both the total serum PSA and the iwPSA, the latter being potentially more representative of tumour burden in case of multiple nodules. Both covariates turned out to independently correlate to DR for both experimental PET/CTs; however, as expected, the estimated DR for total PSA was slightly shallower than the one of iwPSA (see Figure 5, orange vs. green lines). If the curves are (improperly) used to predict the likelihood of finding a local nodule with PET/CT in a different patient population, it should be noted that the predicted DR for a given PSA value needs to be adjusted for the prevalence of positive mpMR in the patient population considered. Moreover, the predicted DR refers to the probability of finding at least one nodule and not all the nodules within a given patient. Given the fact that in our patient population, 10 patients (16.1%) had multiple sites of disease (nine local, one regional), this is not a trivial issue if the purpose of re-staging is the identification of all sites of disease.

## 5. Conclusions

The ability of PET/CT with either Cu or PSMA in the precise localisation of presumed local lesions after RP is poor compared to mpMR, thus not supporting its use as a single imaging method, especially in the context of early sRT. The issue could be logistically overcome with the introduction of PET/MR scanners, although one main open question is whether the use of PSMA is indicated at all in the setting of a population at very low risk of extra prostatic disease [42]; however, PET PSMA has shown prognostic value in this setting [16]. Therefore, whether PSMA should be used to re-stage regional/distant sites before sRT remains controversial, while we provide evidence against its routine use to assess the prostatic bed as an alternative to mpMR.

## Figures and Tables

**Table 1:** Selected patient, tumour, and treatment characteristics.

Characteristic	Stratification	#/Median	%/IQR
Age (years)	Continuum	69.7	65.9–73.6
pT @ RP	pT2 (%)	36	58.06
	pT3a (%)	20	32.26
	pT3b (%)	6	9.68
Margins at RP	R0 (%)	26	41.94
	R1 (%)	36	58.06
PSA @ fail (ng/mL)	Continuum	0.5	0.3–0.7
PSADT (months)	Continuum	9.95	4.80–18.87
Time RP-sRT (months)	Continuum	52.4	25.35–90.95
GGG	1 (%)	5	8.07
	2 or 3 (%)	49	79.03
	4 or 5 (%)	8	12.9
Characteristic	Stratification	#/median	%/IQR
Age (years)	Continuum	69.7	65.9–73.6
pT @ RP	pT2 (%)	36	58.06
	pT3a (%)	20	32.26
	pT3b (%)	6	9.68

Abbreviations: FU, follow up; RP, radical prostatectomy; sRT, salvage radiotherapy; CR, complete response; NR, no response; GGG: Gleason Grade Grouping; In, scan performed in-house; Out: scan performed at outside institution; RP, radical prostatectomy; SD, standard deviation; PSADT, PSA doubling time.

**Table 2:** Location of the lesions at mpMR.

Location	Subsite	#	%
Perianastomotic		42	54.5
	Posterior	27	35.1
	Lateral	9	11.7
	Anterior	6	7.8
Bladder neck		17	22.1
Retrovesical		18	23.4
All		72	100

**Table 3:** Lesions detected within the prostatic fossa by imaging modality.

			Detected Lesions								
	Coregistration		True Positives				False Positives			All	
Imaging Modality			Rigid	Deformable	All		Rigid	Deformable	All		
	# scans	# detectable lesions	#	#	#	% (95%CI)	#	#	#	#	% (95%CI)
mpMR	62	72	65	7	72	100 (94.9–100)	---	---	---	72	100 (94.9–100)
CH/PET	61	71	18	4	22	31.0 (21.5–42.8) *	4	0	4	26	36.6 (26.4–48.2) *
CU/PET	62	72	32	2	34	47.2 (36.1–58.6) *	12	4	16	50	69.4 (58.0–78.9) *
PSMA/PET	58	68	32	5	37	54.4 (42.7–65.7) *	4	2	6	43	63.2 (51.4–73.7) *

* *p* < 0.001 compared to mpMR. Abbreviations: Def, deformable; CI, confidence intervals.

## Data Availability

https://gbox.garr.it/garrbox/index.php/s/epB3nW7lzYnBCM2 (accessed on 31 August 2021).

## Supplementary Materials

The following are available online at https://www.mdpi.com/article/10.3390/cancers13215564/s1, Figure S1: Structure of ^64^Cu-PSMA-617. Figure S2: Co-registration between mpMR and planning CT (plCT). The nodule was contoured on the appropriate phase of the DCE sequence. After co-registration between T2w sequence and plCT, the nodule was transferred to the plCT. Figure S3: CONSORT flow diagram. Table S1: Univariable analysis on DR for each PET/CT tracer.

## References

[1] Tilki D., Mandel P., Schlomm T., Chun F.K., Tennstedt P., Pehrke D., Haese A., Huland H., Graefen M., Salomon G. (2015). External validation of the CAPRA-S score to predict biochemical recurrence, metastasis and mortality after radical prostatectomy in a European cohort. J. Urol..

[2] Stephenson A.J., Shariat S.F., Zelefsky M.J., Kattan M.W., Butler E.B., Teh B.S., Klein E.A., Kupelian P.A., Roehrborn C.G., Pistenmaa D.A. (2004). Salvage radiotherapy for recurrent prostate cancer after radical prostatectomy. Jama.

[3] De Visschere P.J.L., Standaert C., Futterer J.J., Villeirs G.M., Panebianco V., Walz J., Maurer T., Hadaschik B.A., Lecouvet F.E., Giannarini G. (2019). A systematic review on the role of imaging in early recurrent prostate cancer. Eur. Urol. Oncol..

[4] Robertson N.L., Sala E., Benz M., Landa J., Scardino P., Scher H.I., Hricak H., Vargas H.A. (2017). Combined whole body and multiparametric prostate magnetic resonance imaging as a 1-step approach to the simultaneous assessment of local recurrence and metastatic disease after radical prostatectomy. J. Urol..

[5] Kitajima K., Murphy R.C., Nathan M.A. (2013). Choline PET/CT for imaging prostate cancer: An update. Ann. Nucl. Med..

[6] Panebianco V., Sciarra A., Lisi D., Galati F., Buonocore V., Catalano C., Gentile V., Laghi A., Passariello R. (2012). Prostate cancer: 1HMRS-DCEMR at 3T versus [(18)F]choline PET/CT in the detection of local prostate cancer recurrence in men with biochemical progression after radical retropubic prostatectomy (RRP). Eur. J. Radiol..

[7] Valicenti R.K., Thompson I., Albertsen P., Davis B.J., Goldenberg S.L., Wolf J.S., Sartor O., Klein E., Hahn C., Michalski J. (2013). Adjuvant and salvage radiation therapy after prostatectomy: American Society for Radiation Oncology/American Urological Association guidelines. Int. J. Radiat. Oncol. Biol. Phys..

[8] Sanguineti G., Bertini L., Faiella A., Ferriero M.C., Marzi S., Farneti A., Landoni V. (2021). Response on DCE-MRI predicts outcome of salvage radiotherapy for local recurrence after radical prostatectomy. Tumori J..

[9] Connolly J.A., Shinohara K., Presti J.C., Carroll P.R. (1996). Local recurrence after radical prostatectomy: Characteristics in size, location, and relationship to prostate-specific antigen and surgical margins. Urology.

[10] Simone G., Di Pierro G.B., Papalia R., Sciuto R., Rea S., Ferriero M., Guaglianone S., Maini C.L., Gallucci M. (2015). Significant increase in detection of prostate cancer recurrence following radical prostatectomy with an early imaging acquisition protocol with (1)(8)F-fluorocholine positron emission tomography/computed tomography. World J. Urol..

[11] Boellaard R., Rausch I., Beyer T., Delso G., Yaqub M., Quick H.H., Sattler B. (2015). Quality control for quantitative multicenter whole-body PET/MR studies: A NEMA image quality phantom study with three current PET/MR systems. Med Phys..

[12] Giesel F.L., Sterzing F., Schlemmer H.P., Holland-Letz T., Mier W., Rius M., Afshar-Oromieh A., Kopka K., Debus J., Haberkorn U. (2016). Intra-individual comparison of (68)Ga-PSMA-11-PET/CT and multi-parametric MR for imaging of primary prostate cancer. Eur. J. Nucl. Med. Mol. Imaging.

[13] Cornford P., van den Bergh R.C.N., Briers E., den Broeck T.V., Cumberbatch M.G., De Santis M., Fanti S., Fossati N., Gandaglia G., Gillessen S. (2020). EAU-EANM-ESTRO-ESUR-SIOG guidelines on prostate cancer. Part II-2020 update: Treatment of Relapsing and metastatic prostate cancer. Eur. Urol..

[14] Barentsz J.O., Richenberg J., Clements R., Choyke P., Verma S., Villeirs G., Rouviere O., Logager V., Futterer J.J. (2012). European society of urogenital, R. ESUR prostate MR guidelines 2012. Eur. Radiol..

[15] Froemming A.T., Verma S., Eberhardt S.C., Oto A., Alexander L.F., Allen B.C., Coakley F.V., Davis B.J., Fulgham P.F., Hosseinzadeh K. (2018). ACR appropriateness Criteria((R)) post-treatment follow-up prostate cancer. J. Am. Coll. Radiol..

[16] Emmett L., van Leeuwen P.J., Nandurkar R., Scheltema M.J., Cusick T., Hruby G., Kneebone A., Eade T., Fogarty G., Jagavkar R. (2017). Treatment outcomes from (68)Ga-PSMA PET/CT-Informed salvage radiation treatment in men with rising PSA after radical prostatectomy: Prognostic value of a negative PSMA PET. J. Nucl. Med. Off. Publ. Soc. Nucl. Med..

[17] Sharma V., Nehra A., Colicchia M., Westerman M.E., Kawashima A., Froemming A.T., Kwon E.D., Mynderse L.A., Karnes R.J. (2018). Multiparametric magnetic resonance imaging is an independent predictor of salvage radiotherapy outcomes after radical prostatectomy. Eur. Urol..

[18] Dirix P., van Walle L., Deckers F., Van Mieghem F., Buelens G., Meijnders P., Huget P., Van Laere S. (2017). Proposal for magnetic resonance imaging-guided salvage radiotherapy for prostate cancer. Acta Oncol..

[19] Sefrova J., Odrazka K., Paluska P., Belobradek Z., Brodak M., Dolezel M., Prosvic P., Macingova Z., Vosmik M., Hoffmann P. (2012). Magnetic resonance imaging in postprostatectomy radiotherapy planning. Int. J. Radiat. Oncol. Biol. Phys..

[20] Pfister D., Bolla M., Briganti A., Carroll P., Cozzarini C., Joniau S., van Poppel H., Roach M., Stephenson A., Wiegel T. (2014). Early salvage radiotherapy following radical prostatectomy. Eur. Urol..

[21] Mena E., Lindenberg L.M., Choyke P.L. (2019). New targets for PET molecular imaging of prostate cancer. Seminars in Nuclear Medicine.

[22] Nanni C., Zanoni L., Pultrone C., Schiavina R., Brunocilla E., Lodi F., Malizia C., Ferrari M., Rigatti P., Fonti C. (2016). (18)F-FACBC (anti1-amino-3-(18)F-fluorocyclobutane-1-carboxylic acid) versus (11)C-choline PET/CT in prostate cancer relapse: Results of a prospective trial. Eur. J. Nucl. Med. Mol. Imaging.

[23] Perera M., Papa N., Roberts M., Williams M., Udovicich C., Vela I., Christidis D., Bolton D., Hofman M.S., Lawrentschuk N. (2020). Gallium-68 prostate-specific membrane antigen positron emission tomography in advanced prostate cancer-updated diagnostic utility, sensitivity, specificity, and distribution of prostate-specific membrane antigen-avid lesions: A systematic review and meta-analysis. Eur. Urol..

[24] Emmett L., Metser U., Bauman G., Hicks R.J., Weickhardt A., Davis I.D., Punwani S., Pond G., Chua S., Ho B. (2019). Prospective, multisite, international comparison of (18)F-Fluoromethylcholine PET/CT, multiparametric MRI, and (68)Ga-HBED-CC PSMA-11 PET/CT in men with high-risk features and biochemical failure after radical prostatectomy: Clinical performance and patient outcomes. J. Nucl. Med. Off. Publ. Soc. Nucl. Med..

[25] Afshar-Oromieh A., Vollnberg B., Alberts I., Bahler A., Sachpekidis C., Dijkstra L., Haupt F., Boxler S., Gross T., Holland-Letz T. (2019). Comparison of PSMA-ligand PET/CT and multiparametric MRI for the detection of recurrent prostate cancer in the pelvis. Eur. J. Nucl. Med. Mol. Imaging.

[26] Radzina M., Tirane M., Roznere L., Zemniece L., Dronka L., Kalnina M., Mamis E., Biederer J., Lietuvietis V., Freimanis A. (2020). Accuracy of (68)Ga-PSMA-11 PET/CT and multiparametric MRI for the detection of local tumor and lymph node metastases in early biochemical recurrence of prostate cancer. Am. J. Nucl. Med. Mol. Imaging..

[27] Evangelista L., Luigi M., Cascini G.L. (2013). New issues for copper-64: From precursor to innovative PET tracers in clinical oncology. Curr. Radiopharm..

[28] Sproull M., Brechbiel M., Camphausen K. (2003). Antiangiogenic therapy through copper chelation. Expert Opin. Ther. Targets.

[29] Cai Z., Anderson C.J. (2014). Chelators for copper radionuclides in positron emission tomography radiopharmaceuticals. J. Labelled. Comp. Radiopharm..

[30] Peng F., Lu X., Janisse J., Muzik O., Shields A.F. (2006). PET of human prostate cancer xenografts in mice with increased uptake of 64CuCl2. J. Nucl. Med. Off. Publ. Soc. Nucl. Med..

[31] Bartnicka J.J., Blower P.J. (2018). Insights into trace metal metabolism in health and disease from PET: “PET Metallomics”. J. Nucl. Med. Off. Publ. Soc. Nucl. Med..

[32] Paparo F., Peirano A., Matos J., Bacigalupo L., Rossi U., Mussetto I., Bottoni G., Ugolini M., Introini C., Ruggieri F.G. (2020). Diagnostic value of retrospectively fused (64)CuCl2 PET/MRI in biochemical relapse of prostate cancer: Comparison with fused (18)F-Choline PET/MRI, (64)CuCl2 PET/CT, (18)F-Choline PET/CT, and mpMRI. Abdom. Radiol..

[33] Piccardo A., Paparo F., Puntoni M., Righi S., Bottoni G., Bacigalupo L., Zanardi S., DeCensi A., Ferrarazzo G., Gambaro M. (2018). (64)CuCl2 PET/CT in prostate cancer relapse. J. Nucl. Med. Off. Publ. Soc. Nucl. Med..

[34] Cui C., Hanyu M., Hatori A., Zhang Y., Xie L., Ohya T., Fukada M., Suzuki H., Nagatsu K., Jiang C. (2017). Synthesis and evaluation of [(64)Cu]PSMA-617 targeted for prostate-specific membrane antigen in prostate cancer. Am. J. Nucl. Med. Mol. Imaging..

[35] Benesova M., Schafer M., Bauder-Wust U., Afshar-Oromieh A., Kratochwil C., Mier W., Haberkorn U., Kopka K., Eder M. (2015). Preclinical evaluation of a tailor-made DOTA-conjugated PSMA Inhibitor with optimized linker moiety for imaging and endoradiotherapy of prostate cancer. J. Nucl. Med. Off. Publ. Soc. Nucl. Med..

[36] Crocerossa F., Marchioni M., Novara G., Carbonara U., Ferro M., Russo G.I., Porpiglia F., Di Nicola M., Damiano R., Autorino R. (2021). Detection rate of prostate-specific membrane antigen tracers for positron emission tomography/computed tomography in prostate cancer biochemical recurrence: A systematic review and network meta-analysis. J. Urol..

[37] Fonti R., Conson M., Del Vecchio S. (2019). PET/CT in radiation oncology. Semin. Oncol..

[38] Zamboglou C., Fassbender T.F., Steffan L., Schiller F., Fechter T., Carles M., Kiefer S., Rischke H.C., Reichel K., Schmidt-Hegemann N.S. (2019). Validation of different PSMA-PET/CT-based contouring techniques for intraprostatic tumor definition using histopathology as standard of reference. Radiother. Oncol. J. Eur. Soc. Ther. Radiol. Oncol..

[39] Foster B., Bagci U., Mansoor A., Xu Z., Mollura D.J. (2014). A review on segmentation of positron emission tomography images. Comput. Biol. Med..

[40] Zamboglou C., Carles M., Fechter T., Kiefer S., Reichel K., Fassbender T.F., Bronsert P., Koeber G., Schilling O., Ruf J. (2019). Radiomic features from PSMA PET for non-invasive intraprostatic tumor discrimination and characterization in patients with intermediate- and high-risk prostate cancer—a comparison study with histology reference. Theranostics.

[41] Motegi K., Tachibana H., Motegi A., Hotta K., Baba H., Akimoto T. (2019). Usefulness of hybrid deformable image registration algorithms in prostate radiation therapy. J. Appl. Clin. Med. Phys..

[42] Ceci F., Bianchi L., Borghesi M., Polverari G., Farolfi A., Briganti A., Schiavina R., Brunocilla E., Castellucci P., Fanti S. (2020). Prediction nomogram for (68)Ga-PSMA-11 PET/CT in different clinical settings of PSA failure after radical treatment for prostate cancer. Eur. J. Nucl. Med. Mol. Imaging.

