# Supplementary material for: A Prospective Study Assessing the Post-Prostatectomy Detection Rate of a Presumed Local Failure at mpMR with Either 64CuCl2 or 64CuPSMA PET/CT
Source: Cancers (Basel). 2021 Nov 6;13(21):5564. doi: 10.3390/cancers13215564 (PMC8582802; doi:10.3390/cancers13215564)
Supplement: Supplementary file 1 [file cancers-13-05564-s001.zip › cancers-1393919-supplementary.pdf]

cancers

Supplementary Materials: A Prospective Study Assessing the Post-Prostatectomy Detection Rate of a Presumed Local Failure at mpMR with Either $^{64}CuCl_2$ or ^{64}CuPSMA PET/CT

Adriana Faiella, Rosa Sciuto, Diana Giannarelli, Marta Bottero, Alessia Farneti, Luca Bertini, Sandra Rea, Valeria Landoni, Patrizia Vici, Maria Consiglia Ferriero and Giuseppe Sanguineti

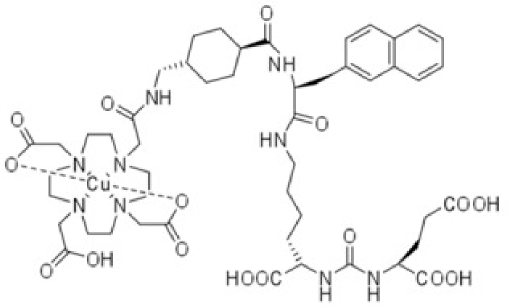

[^{64}Cu]PSMA-617

Figure S1. Structure of ^{64}Cu-PSMA-617. ^{64}Cu was radiolabeled through PSMA-617 (3S,10S,14S)-3-[(naphthalen-2-yl)methyl]-1,4,12-trioxo-1-[(1r,4S)-4-[[2-[4,7,10-tris(carboxymethyl)-1,4,7,10-tetraazacyclododecan-1-yl]acetamido]methyl]cyclo-hexyl]-2,5,11,13-tetraazahexadecane-10,14,16-tricarboxylic acid) provided by ABX, (Radeberg, Germany).

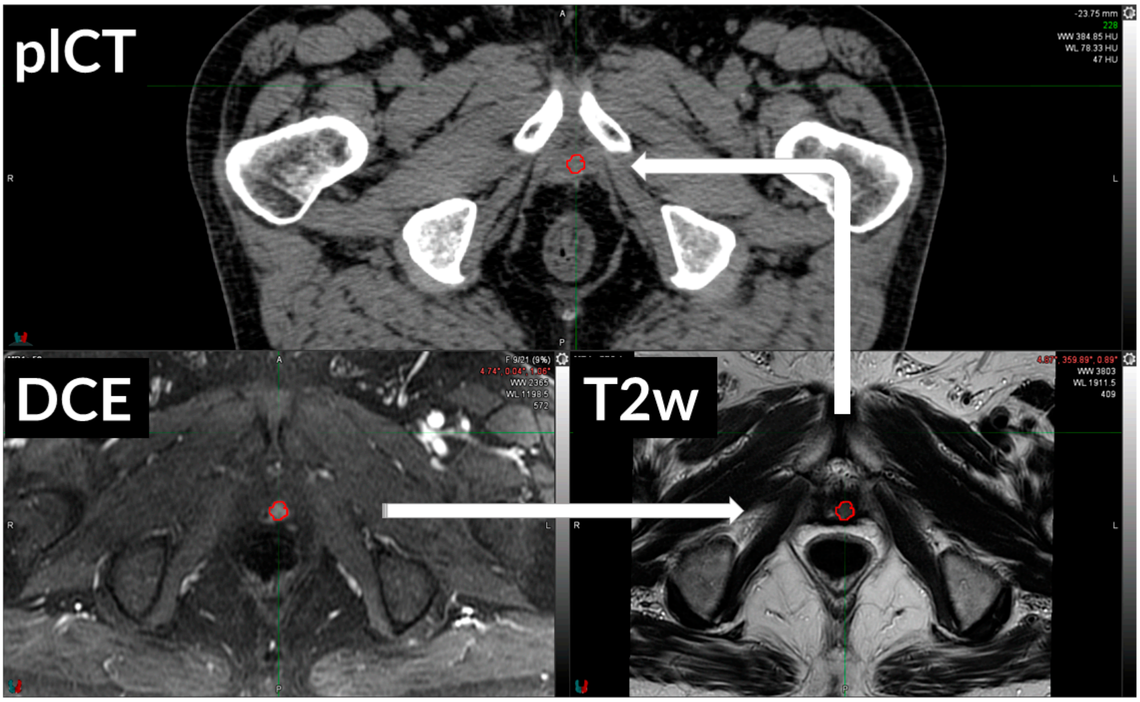

Figure S2. Co-registration between mpMR and planning CT (plCT). The nodule was contoured on the appropriate phase of the DCE sequence. After co-registration between T2w sequence and plCT, the nodule was transferred to the plCT.

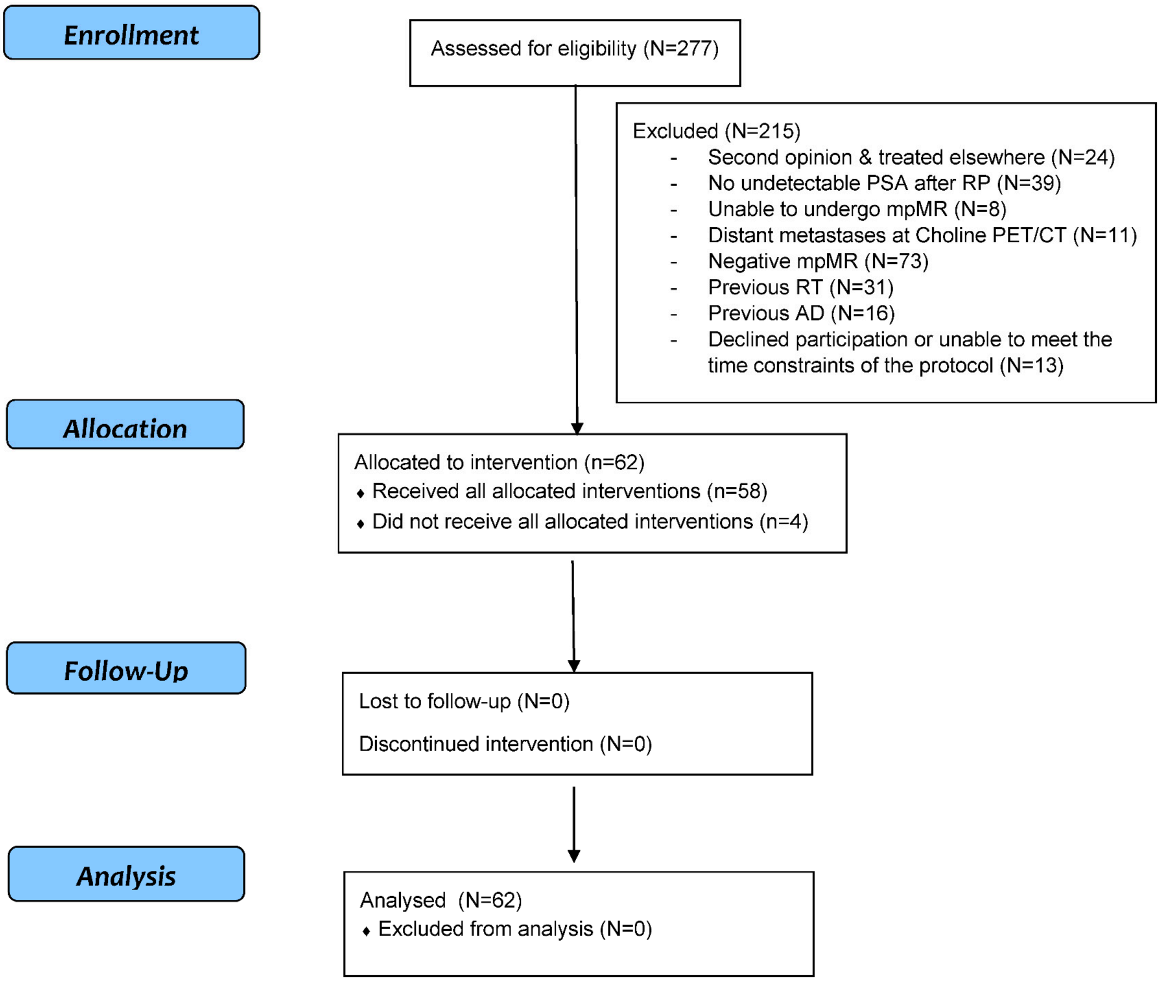

Figure S3. CONSORT flow diagram.

Table S1. Univariable analysis on DR for each PET/CT tracer.

Covariate	Stratification	# nodules	Choline OR (95%CI)	Choline *p*	Cu OR (95%CI)	Cu *p*	PSMA OR (95%CI)	PSMA *p*
**iwPSA (ng/ml)**	Continuum	72	3.282 (1.179–9.138)	0.023	4.200 (1.139–15.482)	0.031	12.715 (1.858–86.996)	0.010
**PSADT (mth)**	Continuum	72	0.996 (0.974–1.019)	0.731	0.968 (0.938–1.000)	0.050	0.988 (0.967–1.009)	0.267
**Volume at mpMR (0.1cc)**	Continuum	72	1.911 (1.319–2.768)	0.001	1.324 (1.059–1.655)	0.014	1.309 (1.045–1.639)	0.019
**Location of the failure**	Anastomotic	39	1		1		1	
	Bladder neck	17	0.667 (0.196–2.273)	0.517	1.069 (0.342–3.344)	0.909	0.869 (0.277–2.728)	0.810
	Retrovescical	16	0.229 (0.045–1.150)	0.073	0.432 (0.126–1.477)	0.181	0.464 (0.141–1.530)	0.207
**GGG**	1–2	33	1		1		1	
	3	29	2.622 (0.860–7.993)	0.090	1.267 (0.464–3.455)	0.644	2.864 (1.020–8.040)	0.046
	4–5	10	1.592 (0.325–7.800)	0.566	2.036 (0.482–8.603)	0.334	4.083 (0.887–18.805)	0.071

Abbreviations: iwPSA, individual weighted PSA; PSADT, PSA doubling time; mpMR, multiparametric MR; GGG, Gleason Grade Group; OR, odds ratio.
